# PARP-1 Expression is Increased in Colon Adenoma and Carcinoma and Correlates with OGG1

**DOI:** 10.1371/journal.pone.0115558

**Published:** 2014-12-19

**Authors:** Tomasz Dziaman, Hubert Ludwiczak, Jaroslaw M. Ciesla, Zbigniew Banaszkiewicz, Alicja Winczura, Mateusz Chmielarczyk, Ewa Wisniewska, Andrzej Marszalek, Barbara Tudek, Ryszard Olinski

**Affiliations:** 1 Department of Clinical Biochemistry, Collegium Medicum, Nicolaus Copernicus University, Karlowicza 24, PO-85-092 Bydgoszcz, Poland; 2 Institute of Biochemistry and Biophysics, Polish Academy of Sciences, Pawinskiego 5a, PO-02-106 Warsaw, Poland; 3 Department of Surgery, Collegium Medicum, Nicolaus Copernicus University, Ujejskiego 75, Bydgoszcz, Poland; 4 Institute of Genetics and Biotechnology, Faculty of Biology, University of Warsaw, Pawinskiego 5a, PO-02-106 Warsaw, Poland; 5 Department of Clinical Pathomorphology, Collegium Medicum, Nicolaus Copernicus University, Sklodowskiej-Curie 9, PO-85-092 Bydgoszcz, Poland; 6 Department of Oncologic Pathology, University of Medical Sciences and Wielkopolskie Oncology Center, Garbary 15, PO-61-868 Poznan, Poland; Sapporo Medical University, Japan

## Abstract

The ethiology of colon cancer is largely dependent on inflammation driven oxidative stress. The analysis of 8-oxodeoxyguanosine (8-oxodGuo) level in leukocyte DNA of healthy controls (138 individuals), patients with benign adenomas (AD, 137 individuals) and with malignant carcinomas (CRC, 169 individuals) revealed a significant increase in the level of 8-oxodGuo in leukocyte DNA of AD and CRC patients in comparison to controls. The counteracting mechanism is base excision repair, in which OGG1 and PARP-1 play a key role. We investigated the level of PARP-1 and OGG1 mRNA and protein in diseased and marginal, normal tissues taken from AD and CRC patients and in leukocytes taken from the patients as well as from healthy subjects. In colon tumors the PARP-1 mRNA level was higher than in unaffected colon tissue and in polyp tissues. A high positive correlation was found between PARP-1 and OGG1 mRNA levels in all investigated tissues. This suggests reciprocal influence of PARP-1 and OGG1 on their expression and stability, and may contribute to progression of colon cancer. PARP-1 and OGG1 proteins level was several fold higher in polyps and CRC in comparison to normal colon tissues. Individuals bearing the *Cys326Cy*s genotype of OGG1 were characterized by higher PARP-1 protein level in diseased tissues than the *Ser326Cys* and *Ser326Se*r genotypes. Aforementioned result may suggest that the diseased cells with polymorphic OGG1 recruit more PARP protein, which is necessary to remove 8-oxodGuo. Thus, patients with decreased activity of OGG1/polymorphism of the OGG1 gene and higher 8-oxodGuo level may be more susceptible to treatment with PARP-1 inhibitors.

## Introduction

Oxidative damage to DNA has often been blamed as a possible basis for the physiological changes associated with cancer [1]–[3] and 8-oxo-7,8-dihydroguanine (8-oxoGua), one of the oxidatively modified DNA bases, is a typical biomarker of the damage. Moreover, many observations indicate a direct correlation between 8-oxoGua formation and carcinogenesis *in vivo* [1], [2].

To counteract the deleterious effect of oxidatively damaged DNA, all organisms developed several DNA repair pathways. Excision of 8-oxoGua from DNA is accomplished mainly by base excision repair (BER) and the major enzyme catalyzing the removal of 8-oxoGua is the OGG1 DNA glycosylase/AP lyase [4], [5]. Another protein which plays a key regulatory role in BER is PARP-1, which is a molecular sensor of DNA breaks [6]. Moreover, PARP-1 is activated in response to DNA damage including oxidatively modified nucleotides [7], [8]. Both enzymes may, in addition, contribute to cancer progression, regulating the expression of critical genes. PARP-1 may stimulate transcription of the *c-MYC* gene, by converting the guanine-quadruplex structure in the human *c-MYC* gene's promoter into B-DNA, and thus facilitating access to this promoter for transcription factors [9]. OGG1, in turn, facilitates transcription of genes regulated by c-MYC. LSD1 histone methylase oxidizes G to 8-oxoGua within promoters of c-MYC regulated genes. Subsequent recruitment of OGG1, which excises 8-oxoGua and incises DNA at the site of the damage causes promoter relaxation and stimulates transcription [10].

In recently published paper we have demonstrated the existence of oxidative stress/DNA damage in colorectal carcinoma patients (CRC) and in patients with precancerous condition - benign adenoma (AD) [11]. This was accompanied by increased 8-oxoGua excision rate in blood leukocytes of CRC patients, and high frequency of OGG1 glycosylase Cys326Cys genotype among CRC patients but not among AD individuals and healthy controls. However, despite the higher excision rate, 8-oxodGuo level in DNA of blood leukocytes was elevated both in CRC patients and AD individuals in relation to healthy volunteers. Seemingly, the higher 8-oxoGua excision rate was insufficient to counteract the increased DNA damage and/or also other factors regulating 8-oxodGuo level in leukocyte DNA.

Several papers reported that PARP-1 is overexpressed in various human malignancies [12]–[15]. Moreover, it was demonstrated that PARP-1 plays a role in colon cancer development [16]–[18] since its expression was significantly higher in colon cancer and was correlated with tumor size and histopathology [18]. Recent clinical trials demonstrated that PARP-1 inhibitors may be used against different types of cancers, as reviewed in [19]–[21]. It has also been shown demonstrated that direct interaction of PARP-1 and OGG1 is involved in the repair of oxidatively damaged DNA [8]. Moreover, it has been suggested that in the absence of OGG1 cells are sensitized to PARP inhibitors [8].

Other studies showed that mRNA levels of *OGG1* and *ERCC1* genes are significantly increased in colon lesions in the adenoma-carcinoma pathway, and that this increase was higher in severe lesions, namely severe adenomas and carcinomas, than in mild ones [22]. Moreover, the expression of DNA repair genes was highly correlated, and depended mostly on variations in genetic construction of individuals (individual variations were significantly higher than seasonal) [22]. Interestingly, carriers of the Cys326Cys genotype had higher level of OGG1 mRNA than carriers of the wild type enzyme [23].

To have a better insight into the relationship between oxidatively damaged DNA/repair and PARP-1 and their involvement in cancer development, we presently investigated the mRNA/proteins expression of PARP-1 and OGG1 and the 8-oxodGuo level in DNA of normal and diseased colon tissues and in leukocytes of CRC patients and individuals developing benign adenomatous polyps as well as in leukocytes of control healthy subjects.

## Materials and Methods

### Ethics statement

The study was conducted in accordance with the Declaration of Helsinki, and the protocol was approved by the medical ethics committee of Collegium Medicum, Nicolaus Copernicus University, Bydgoszcz, Poland. All participants of the study signed informed consent.

### Study group

The study was performed in three groups. The control group (H) of healthy volunteers (n = 138) comprised 64 males and 74 females (median age 55±8.1 years for men and 52±8.1 for women). The adenoma (AD) patient group (n = 137) comprised 69 males and 68 females (median age 64±11.8 years for men and 61±9.9 for women). The carcinoma (CRC) patient group (n = 169) comprised 88 males and 81 females (median age 63±11.9 years for men and 64±14.3 for women). The groups were chosen in such a way that the following criteria were matched: eating habits, age, body weight and smoking status. All the subjects, when recruited to the study, filled in the questionnaire concerning demographic data, smoking, diet and medical history. Interviewees were asked to estimate the average frequency of consumption of various dietary items in the year proceeding the interview. The majority of them reportedly consumed 3 servings of fruit and vegetables and about 250 g of meat and fat per day. To make the group even more homogenous, the subjects who reported the extreme consumption, as well as those who reported supplementation within the last month were excluded from the study. The questionnaire was administered by the team physician (Dr. Banaszkiewicz). The control group was chosen to maximally match the patient groups and adenoma individuals by age, sex, diet (consumption of fat, carbohydrates and vitamin intake), body weight, and smoking status. There were no differences between the studied groups in relation to body mass and stature of men and women. All participants were Caucasians and there were no relatives among them. All individuals participating in the study were recruited through the hospital (Collegium Medicum, Nicolaus Copernicus University, Bydgoszcz, Poland) and were examined by colonoscopy.

### Immunohistochemistry

Blocks with paraffin embedded pieces of tissue were cut into 4.0 µm thick sections using microtome Accu-Cut SRM 200 (Sakura Finetek Europe B.V., Netherlands), placed on SuperFrost glass slides (Thermo Scientific, USA) and dried at 58°C for 1 h. For immunohistochemical analysis the paraffin tissue sections were dewaxed in xylene and gradually hydrated in decreasing series of alcohol (100, 96, 90, 70%) and finally brought to water. After antigens retrieval (Dako Target Retrieval solution high pH; catalogue number K8000; Dako) at 95°C for 20 min and blocking endogenous peroxidases activity in 3% H_2_O_2_ at room temperature for 10 min, the slides were incubated in PBS-Tween buffer containing 5% BSA to block nonspecific staining also at room temperature for 10 min. Subsequently, the sections were incubated with primary rabbit monoclonal antibody against PARP (catalogue number ab32138; abcam) or rabbit polyclonal antibody directed to hOGG1 (catalogue number NB100-106SS; Novus Biologicals, USA). The incubation with primary antibodies (diluted, respectively, 1∶50 and 1∶500 in PBS containing 1.0% BSA) was carried out overnight at 4°C in a moist chamber. After being washed twice with PBS-Tween, the slides were incubated using the anti-rabbit EnVision peroxidase detection system (catalogue number K4011 Dako) for 1 h at room temperature. For chromogenic detection DAB detection kit was used (catalogue number SM 803, SM827; Dako). After rinsing in distilled water, the sections were counterstained with haematoxylin, dehydrated in increasing grades of alcohol (70, 90, 96, 100%) and finally mounted with Shandon Consul Mount (Thermo Scientific). Negative controls were prepared using the same procedure except that the primary antibody was replaced with 1% BSA in PBS.

### Morphometric analysis

Specimens were examined with light microscope (Eclipse E800, Nikon) equipped with cooled CD camera (Nikon Digital Sight DS-5Mc, Germany) driven by NIS Elements F 3.0 software (Nikon, Germany). The expression levels of antigens were evaluated by staining intensity of the cell's compartments after immunohistochemical detection of the analyzed antigens using the method described by Remmele and Stegner [24]. That scoring system (IRS) took into account the intensity of the colour reaction (SI) and the percentage of positive cells (PP) within the five microscope fields at the magnification of 200 x. The final score represented a product of scores (SI × PP) and ranged from 0 to 12 points (low reaction: 1 to 2 points, average reaction: 3 to 4 points, intense reaction: 6 to 12 points). The images were prepared using CorelDraw12 software.

### Isolation of leukocytes from venous blood

Blood samples were taken from patients and controls in the morning before breakfast in the Clinical Units of Collegium Medicum Nicolaus Copernicus University in Bydgoszcz. Blood samples (18 ml) were carefully applied on top of Histopaque 1119 solution (Sigma-Aldrich Inc.; St.Louis, MO, USA) and leukocytes were isolated by centrifugation according to the manufacturer's protocol.

### Measurement of oxidative stress biomarkers in tissues

All DNA modifications were analyzed using high performance liquid chromatography/electrochemical detection (HPLC/EC) technique. Expression of repair proteins was analyzed by QPCR or Western methods. Antibodies used for Western blot were the following: primary rabbit monoclonal antibody anti PARP-1 (E-102, abcam, catalogue number ab32138) or anti Lamin A/C (rabbit polyclonal IgG sc-7150) and Goat- anti-rabbit polyclonal IgG-HRP (sc-2004, Santa Cruz) as secondary antibody, anti-OGG1/2 goat polyclonal IgG (Santa Cruz Biotechnology) and donkey anti-goat IgG-HRP (Santa Cruz Biotechnology). All methods were described in detail elsewhere [11]. Polymorphism of Ser326Cys OGG1 glycosylase was identified by multitemperature single strand conformation polymorphism method (MSSCP, Biovectis) as described in [11].

### RNA extraction and cDNA synthesis

Total RNA was isolated from frozen leukocytes using the TRIzol reagent (Invitrogen, Carlsbad, CA, USA). The quality of total RNA was checked by formaldehyde-agarose gel electrophoresis, and for further analyses only RNA samples with clearly distinguished 18S and 28S ribosomal RNAs and no visible RNA degradation were used. Total RNA (2 µg) from each sample was used to generate cDNA using the High Capacity cDNA Reverse Transcription Kit (Applied Biosystems by Life Technologies; Carlsbad, CA, USA) with random primers.

### Real-time PCR using SYBR-Green chemistry

Real-time PCR assays were carried out on a Roche LightCycler480 System apparatus. Each reaction was carried out in 10 µl mixture of the AmpliQ Real-Time PCR SYBR Green KIT containing: 1× Taq polymerase buffer (without MgCl_2_), 3 mM MgCl_2_, 0.01% Tween 20, 0.8% glycerol, 5% DMSO, 0.5 ng/µl acetylated BSA, dATP, dCTP, dGTP and dTTP – 400 µM each, 1× concentrated reference dye ROX, 1∶40000 diluted SYBR Green, 0.625 U of Taq polymerase, forward and reverse primers, 400 µM each, and cDNA template. The time -temperature program was as follows: 95°C for 3 min as initial denaturation step followed by 45 cycles consisting of a denaturation step at 95°C for 15 s, primer annealing at 60°C for 15 s and an extension step at 72°C for 1 min. Fluorescence was read during the extension step of each cycle. Melting-point temperature analysis was performed in the range of 60 to 95°C, with temperature increments of 0.33°C. Background range and threshold for C_t_ evaluation in each experiment were adjusted manually.

The primers were designed using the Primer Express program (Applied Biosystems; Foster City, CA, USA). The following PCR sense and antisense primers (sequence 5'->3' respectively) were used:

- *PARP-1* (TCAGCCTCCTTGCTACAGAGG and GGTCGTTCTGAGCCTTTAGGG),
- *OGG1* (CAGCTCCACTGCACTGTGTACC and TCGCACACCTTGGAATTTCTG),
- *c-MYC* (CCTACCCTCTCAACGACAG and TCTTGTTCCTCCTCAGAGTC),
- *PMM1* (CGCCTTCCTGCAGAAGCTAC and TCTGCTTGGAGAGCAGTCGTC),
- *RPII* (TTGGCTTCAAGCACCGGAC and ATCCAGTCTCAGCAGTCTTGACAG).

Before use the primers were tested for equal efficiency of the PCR reaction by the 2^-ΔΔCt^ method validation [25]. Each experiment involved measurement of C_t_ values for three amounts of the template, each in duplicate. The template amounts per sample were as follows: 30, 120 and 480 ng for all the genes. The efficiency (values between 0 and 1) of the qPCR reaction with each primer pair was calculated, and subsequently used to calculate the ratio of each studied gene to the reference gene. Only efficiency values ≥0.95 were accepted.

For each cDNA sample four reactions were carried out using two template amounts of 10 and 40 ng, each in duplicate. The quality of results was evaluated on the basis of expected C_t_ differences between the two cDNA amounts as well as product melting curves. A few rare outlying results were omitted in the calculations. For each gene the amounts of cDNA were chosen individually (if possible, the same for all genes) to obtain C_t_ values in the range between 14 and 34 cycles.

The results were calculated with normalization of C_t_ values to mean C_t_ value for the *PMM1* and *RPII* reference genes as described [26].

### Statistical analyses

To summarize the data the following descriptive statistics: median, first quartile (Q_1_), third quartile (Q_3_), minimum and maximum values, were calculated for each quantitative variable. The resulting groups comprised similar number of patients.

For the statistical analysis the STATISTICA (version 10.0) computer software (StatSoft, Inc) was used. For normal distribution, variables were analyzed by the Kolmogorov–Smirnov test with Lillefor's correction. For variables with non-parametric distribution U Mann–Whitney's test was carried out; for variables with normal distribution - Student-t test. Statistical significance was considered at p<0.05.

## Results

### Expression of PARP-1 and OGG1 mRNA in leukocytes and colon tissues

**Figure 1:**
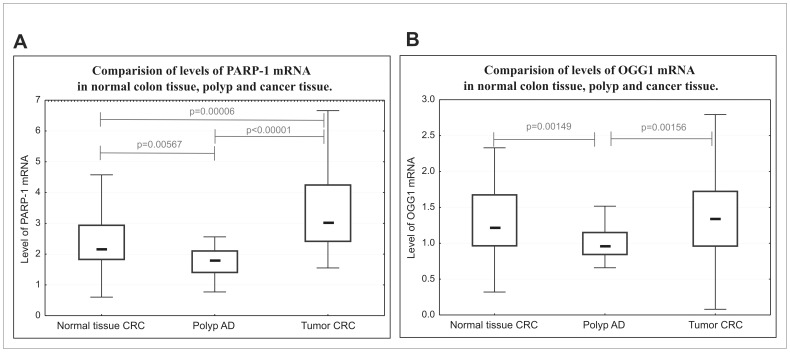
Level of PARP-1 (A) and OGG1 (B) mRNA in normal colon tissue (n = 60), polyp (n = 24) and cancer tissue (n = 60). Center mark in the box indicates the medians of the samples. The length of each boxes (IQR, interquartile range) represents the range within which the central 50% of the values fell, with the vertical edges placed at the first and third quartiles. Whiskers show variability outside the upper and lower quartiles. *P* was obtained with the Mann-Whitney test.

**Figure 2:**
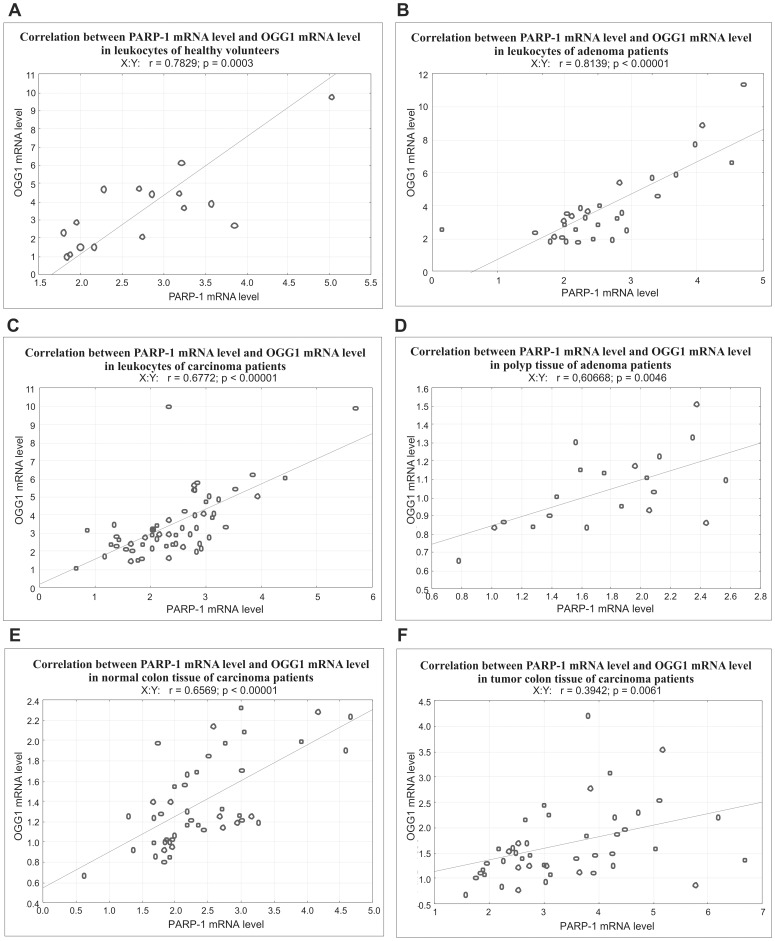
Correlation between PARP-1 and OGG1 mRNA levels in leukocytes of healthy volunteers (A), adenoma patients (B) and carcinoma patients (C), as well as in polyp tissue (D), normal colon (E) and tumor (F).

In leukocytes of healthy controls, and AD and CRC patients the level of PARP-1 and OGG1 mRNA was similar (see S1 Table). However, PARP-1 and OGG1 mRNAs retained the tendency to be lower in benign adenoma tissue than in colon tissue without histological changes (normal colon) of CRC patients (p = 0.00567 and p = 0.00149 respectively) and lower than in colon tumor tissue (p<0.00001 and p = 0.00156 respectively) - Fig. 1. In order to validate mRNA measurements, we also estimated mRNA level of c-MYC, which is one of the targets in Wnt pathway. This pathway is activated at the very early stage of colon carcinogenesis, namely 50–70% of early and medium adenomas carry the mutation in the *APC* gene [27]. The results show clearly a significant increase in c-MYC mRNA level both in adenomas and carcinomas (S2 Table), thus validating our estimations of mRNA level. Unfortunately, the material obtained from adenoma patients did not contain well distinguished normal colon tissue which could be seen in histological sections as marginal tissue, and would enable to measure PARP-1 and OGG1 proteins in adenoma and normal colon from the same patient. We were therefore unable to compare mRNA level by qPCR in normal and adenoma tissues from AD patients. The expression of mRNA of both enzymes was the highest in colon tumors. However, only PARP-1 mRNA in tumor significantly exceeded that in normal tissue (p = 0.00006, Fig. 1), for OGG1 the differences were statistically insignificant (p = 0.24). This finding suggests that PARP-1 mRNA expression is a better indicator of colon cancer development, than OGG1 mRNA. High positive correlation was found between mRNA level of PARP-1 and OGG1 (Fig. 2) in leukocytes of healthy volunteers (r = 0.7829), AD patients (r = 0.8139), and CRC patients (r = 0.6772), as well as in all colon tissues of CRC patients (Fig. 2). This may suggest that both genes are under the same transcriptional control.

### PARP-1 and OGG1 protein is overexpressed in colon adenomas and carcinomas

**Figure 3:**
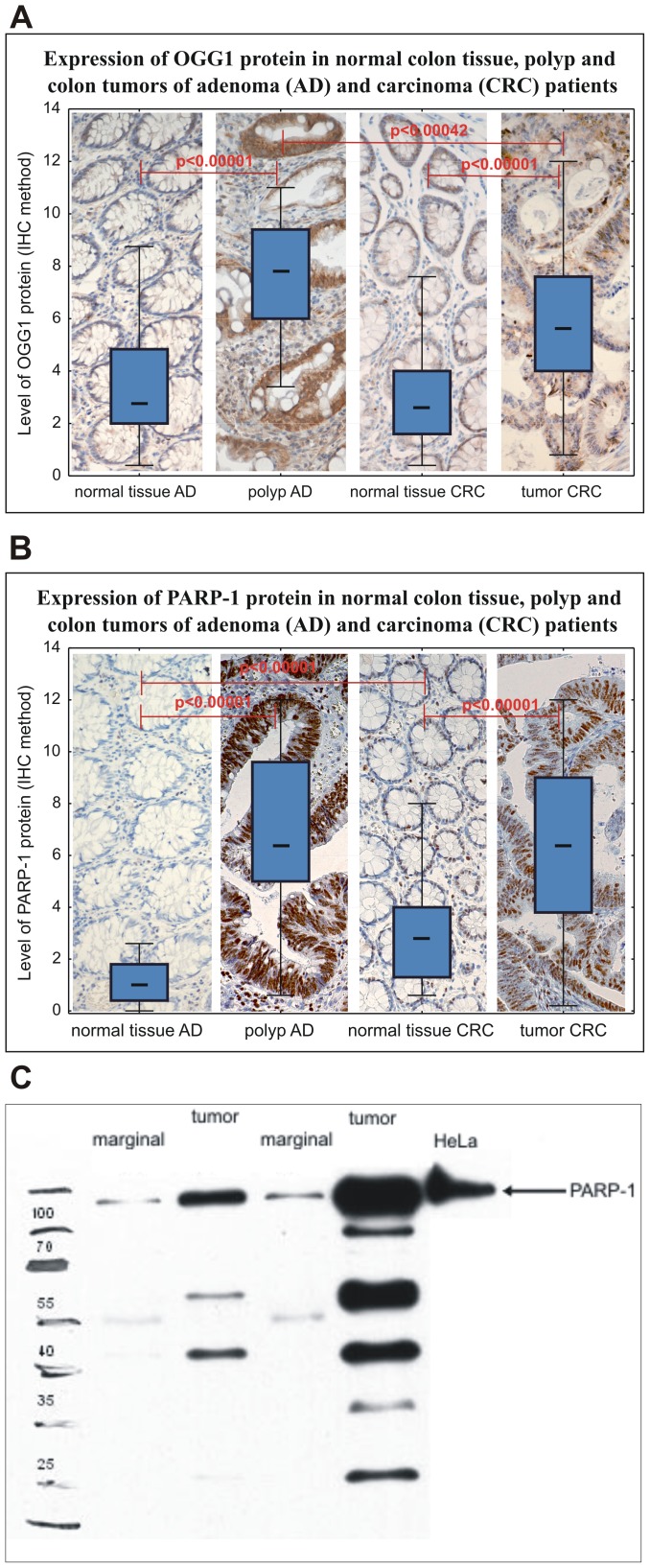
Comparison of the expression of OGG1 (A) and PARP-1 (B) protein in normal colon tissue, polyp and cancer tissue of adenoma (AD, n = 68) and carcinoma (CRC, n = 103) patients. Immunohistochemical detection in paraffin embedded sections stained with hematoxylin and eosin. Center mark in the box indicates the medians of the samples. The length of each box (IQR, interquartile range) represents the range within which the central 50% of the values fell, with the vertical edges placed at the first and third quartiles. Whiskers show variability outside the upper and lower quartiles. *P* was obtained with the Mann-Whitney test. Representative examples of the levels of PARP-1 protein in tissues of CRC patients determined by Western analysis. The analysis was performed on tumor and normal tissues of 41 CRC patients (C).

Immunohistochemical studies have shown that in colon adenomas and carcinomas the amount of proteins identified by anti-PARP-1 antibody was significantly higher than in surrounding colon tissue without histological changes (Fig. 3B). Morphometric measurements of colon sections have shown that colon tumor tissue contained about three times greater amount of PARP-1 protein than unaffected colon tissue (mean 6.4, range 3.80–9.0 in tumor *versus* 2.4, range 1.0–4.0 in normal colon, p<0.00001, Fig. 3B). This difference was even greater in adenomas. Adenoma tissue contained almost 10 times greater amount of PARP-1 protein than normal colon (6.4, range 5.0–9.0 in adenoma *versus* 0.66, range 0.2–1.2 in normal colon, p<0.00001, Fig. 3B). Interestingly, in normal colon tissue of AD patients the amount of PARP-1 protein was lower than in normal colon of CRC patients (0.66, range 0.2–1.2 in AD patients *versus* 2.4, range 1.0–4.0 in CRC patients, p = 0.00001, Fig. 3B). This could suggest that higher expression of PARP-1 could predispose to further development of cancer. It should be noted that only some adenomas develop into carcinomas, and most AD patients will never develop cancer. We wanted to confirm the results obtained by immunohistochemistry by a standard Western blot using the same antibody as for colon sections (Fig. 3C). Western analysis confirmed an increased amount of PARP-1 in colon tumors in relation to normal colon (Fig. 3C), but also revealed that anti-PARP-1 antibody reacted also with other proteins, which were particularly abundant in tumor tissue. Some of the additional bands could be the products of apoptotic PARP-1 cleavage by caspases 3 and 7 into 89 and 24 kDa products [28]. Other bands could be derived from PARP-1 cleavage by metalloproteinases. Kwan and coworkers [29] have shown that incubation of purified bovine PARP-1 with human metalloproteinase 2 (MMP-2) resulted in the loss of the 116 kDa band of intact PARP-1 and the appearance of 66 and 45 kDa products of PARP-1 cleavage by MMP2. The protein band of 116 kDa corresponding to intact PARP-1 protein measured in relation to Lamin A/C was 2.13 times larger in tumor than in normal tissue. Thus, these results were similar to those obtained in immunohistochemical examination (3 times greater amount of PARP-1 in tumor than in normal colon), although the differences between normal and diseased tissues might be slightly overestimated in immunohistochemical examination.

**Figure 4:**
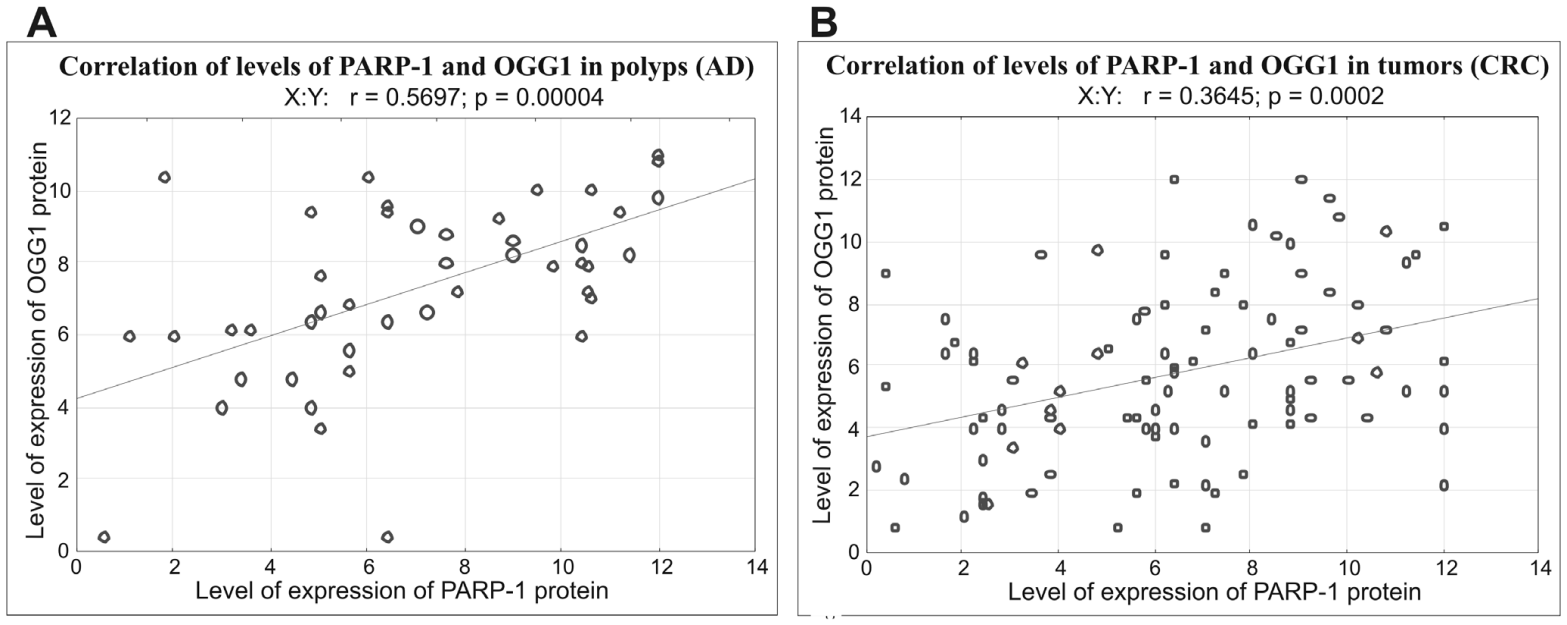
Correlation between levels of expression of PARP-1 and OGG1 protein in dysplastic cells of AD (A) and CRC (B) patients.

**Figure 5:**
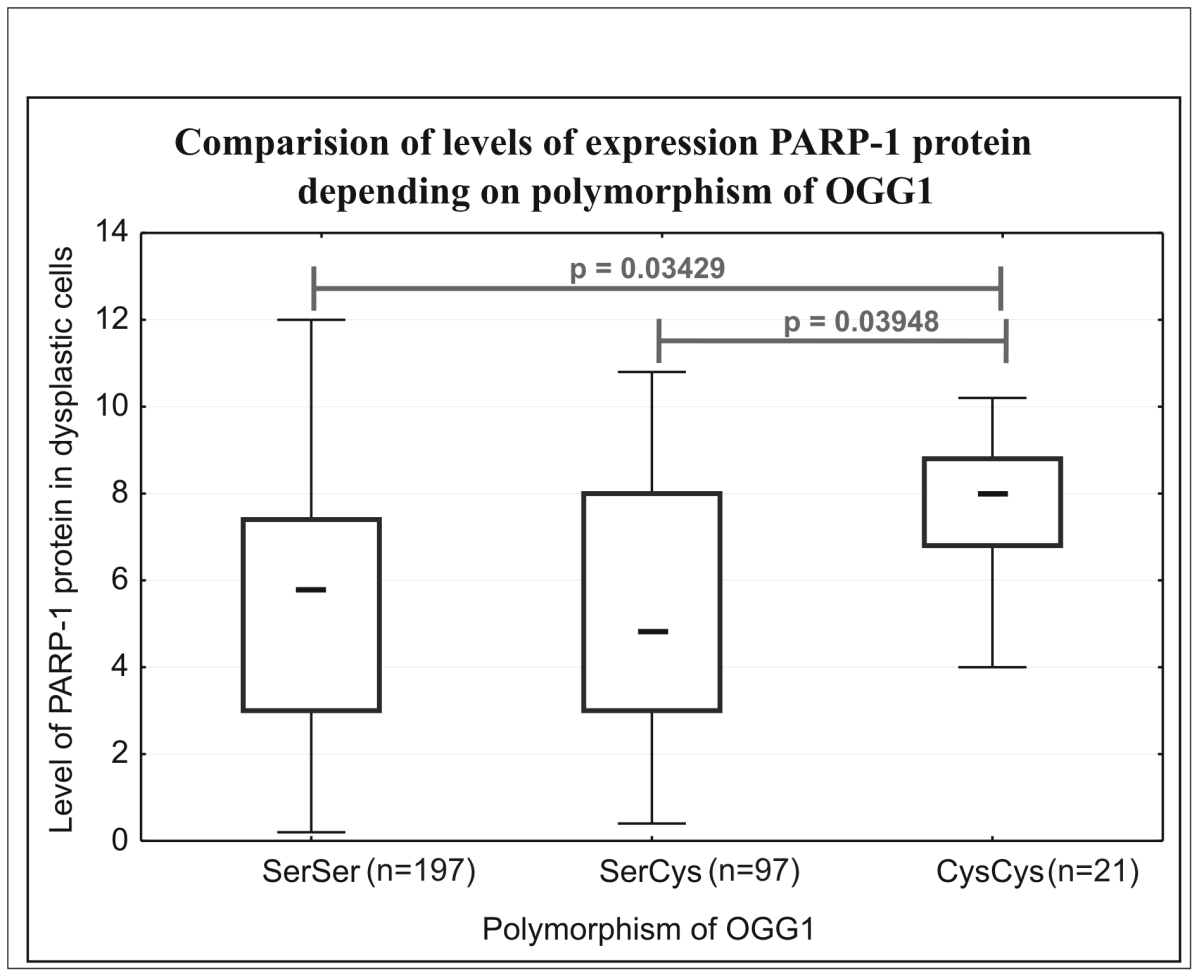
Comparison of the level of PARP-1 protein expression in relation to OGG1polymorphism. Center mark in the box indicates medians for samples. The length of each box (IQR, interquartile range) represents the range within which the central 50% of the values fell, with the vertical edges placed at the first and third quartiles. Whiskers show variability outside the upper and lower quartiles. *P* was obtained with the Mann-Whitney test.

The amount of OGG1 protein was also higher in carcinoma and adenoma tissues than in normal colon (5.60, range 4.0–7.60 in carcinoma *versus* 2.50, range 1.60–4.0 in normal colon, p<0.00001, and 7.80, range 6.10–9.30 in adenoma tissue *versus* 2.29, range 1.25–5.20 in normal colon) although these differences were not as big as for PARP-1. In contrast to PARP-1, the amount of OGG1 in normal colon did not differ between AD and CRC patients (Fig. 3A). A correlation between PARP-1 and OGG1 proteins content was found in adenoma tissues (r = 0.5697) and tumor tissues (r = 0.3645) (Fig. 4). However, no such correlation was observed in normal tissues – see S1 Fig. A very intriguing association was found between *OGG1 Ser326Cys* polymorphism and PARP-1 protein level in tumors and polyps of CRC and AD patients. The patients of *OGG1 Cys326Cys* genotype had significantly higher PARP-1 protein level than those with *Ser326Cys* and *Ser326Ser* genotype (p = 0.03948 and 0.03429, respectively, Fig. 5, S1 Fig.). OGG1 326 Cys variant is a protein with decreased enzymatic activity, revealed only upon oxidative stress [4]. Whether the diseased cells with polymorphic OGG1 recruit more PARP protein which is necessary to remove 8-oxodGuo remains to be further studied.

No correlation was found between tumor staging and PARP1/OGG1expression.

### Oxidative stress during CRC development

We have previously shown that 8-oxo-7,8-dihydroguanine is an efficient predictor of survival for colon cancer patients [30] and that oxidative stress gradually increases in individuals at different stages of colon cancer development [11]. Our data demonstrated about 30% increase in 8-oxodGuo level in peripheral leukocytes of AD and CRC patients in relation to healthy controls. Here, we show that such a tendency was not retained for diseased tissue [11]. No difference in DNA 8-oxodGuo level was found between normal colon tissue of CRC patients, benign adenomas and malignant carcinomas. In contrast, a small, although statistically non-significant decrease was found in consecutive stages of CRC development, namely in polyp and tumor tissues in relation to unaffected/normal colon of CRC patients (S3 Table). This might reflect increased 8-oxoGua elimination from DNA, which would be consistent with the significant increase in OGG1 and PARP-1 protein in adenoma and carcinoma tissues in relation to normal colon tissue of CRC patients. However, no correlation was found between PARP-1 or OGG1 level and 8-oxodGuo content in these tissues (except for leukocytes – OGG1 mRNA *vs* 8-oxodGuo: r = 0.4952, p = 0.0046). This might derive from the contribution of other repair systems to control of 8-oxodGuo level in DNA, namely of MTH phosphohydrolase and the mismatch repair system [31].

## Discussion

There is increasing evidence that most human cancers contain large numbers of mutations [32]. This in turn suggests that they are generated continuously during tumor progression. Endogenous cellular processes (oxidative phosphorylation, peroxisomal fatty acid metabolism, cytochrome P-450 reactions or “respiratory burst” of phagocytic cells) are efficient sources of harmful ROS that may be responsible for oxidatively modified DNA bases and may serve as a source of mutations that initiate carcinogenesis. Since severe oxidative stress is also characteristic for advanced stages of cancer development, these modifications may also serve as an efficient source of mutations during tumor progression.

Although on the basis of available experiments there is little room for doubt that oxidative DNA damage plays some role in the pathogenesis of cancer, there is still no evident relationship between the measured DNA damage and the rate of mutation and cancer. One of the factors which may be associated with predisposition to cancer is deregulation of DNA damage repair, which may in turn be also linked to the effectiveness of anticancer therapy. The major pathway for repair of oxidative damage to DNA is base excision repair (BER) with OGG1 and PARP-1 being important enzymes responsible for removal of 8-oxoGua. Moreover, it has recently been shown that OGG1 binds PARP-1 directly and that formation of this complex is enhanced by oxidative stress [8].

In our work the oxidatively damaged DNA (reflected by 8-oxodGuo level) together with the enzymes involved in repair of the damage were analyzed in diseased and normal (marginal) tissues taken from patients bearing benign adenomas (AD) and colon cancer (CRC) and in leukocytes from the patients as well as from healthy subjects (control group).

We have observed good and highly statistically significant correlation between mRNA expression of OGG1and PARP-1 in investigated tissues: in leukocytes of all groups (control, patients with adenoma and CRC) and in normal tissues as well as in adenoma and CRC – Fig. 2. The good correlation may indicate that both genes, which are the main players in repair of oxidatively damaged DNA, are at least partially expressed in response to the same stimuli – oxidative stress. Indeed, several studies have demonstrated that the expression of OGG1 [33] and PARP-1 [34]–[37] is enhanced as a result of oxidative stress, and our recently published paper has shown increased oxidative stress in colon adenoma and carcinoma patients [11]. However other factors may differently regulate the expression of OGG1 and PARP-1 (i.e. SSB may strongly induce PARP-1 expression). We understand that further studies are necessary to confirm our findings.

The results also showed a several fold higher level of PARP-1 and OGG1 proteins in cancerous tissues than in normal ones (Fig. 3). Moreover, the differences were even more pronounced in the case of benign adenomas, the earliest premalignant lesions, that may subsequently progress to invasive carcinomas. Protein and mRNA levels of PARP-1 in adenoma and carcinoma tissues differ; the mRNA level is lower in adenoma than in carcinoma, while the protein level is similar in both tissues (Figs. 1 and 3). A similar inconsistency was found in the studies of Paz-Elizur and co-workers [38] who showed poor correlation between OGG1 activity and mRNA level in human blood cells. This may suggest the importance of factors other than mRNA expression in the control of protein level and activity, e.g. protein stability or the rate of its degradation. One of the reason of the above mentioned inconsistency concerning mRNA and protein level may be field cancerization. It is possible that relatively high values of mRNA expression in normal CRC tissue when compared with polyp AD is a result of field cancerization. Although histopathological analyses has failed to detect tumor cells in marginal/normal tissue, the molecular assays demonstrated presence of cells clonally related to the tumor (field cancerization) [39]. Moreover, the field cancerization in the case of CRC colon marginal tissue may involve patches measuring up to 10 cm [40]. Therefore, it is likely that relatively large marginal tissue from which mRNA was isolated comprised aforementioned field cancerization. However, in the case of immunohistochemical analyses, the most distant, small part of normal CRC tissue was analysed, what in turn, minimized a likelihood of the presence of field defects.

The higher expression of DNA repair proteins in cancer tissue is in line with the about 50% lower level of 8-oxodGuo in DNA isolated from cancerous tissue in comparison with surrogate tissue - leukocytes of CRC patients (see S3 Table). Interestingly, we have recently demonstrated that the εAde and εCyt (other mutagenic DNA adducts induced by oxidative stress) excising activities were much higher in cancerous tissue than in the surrounding normal colon in agreement with the 2–3 times lower εdA and εdC adducts level [41].

An increase in oxidative stress during colon carcinogenesis has been demonstrated by us [41], [42] and other groups [43], [44]. The important question is why in the patients groups the level of oxidatively damaged DNA marker is decreased in colon cancer and adenoma tissues despite oxidative stress symptoms, on the level of the whole organism? As mentioned above, the decrease in 8-oxodGuo level in cancerous tissue may be explained by the increased expression of repair enzymes involved in removal of this modification. Therefore, a more relevant question concerns the higher expression of OGG1 and PARP-1 in the diseased tissues from AD and CRC patients.

Induction of DNA repair genes may occur at the early stage of the carcinogenic pathway, and may be caused by increased oxidative stress. Indeed, it was demonstrated that some tumor cell lines can produce significant levels of H_2_O_2_ without exogenous stimulation [1]. It has also been shown that antioxidant enzymes activity/expression may be downregulated in cancerous and precancerous cells [45]. These conditions may be directly responsible for oxidative stress, which in turn may lead to OGG1 and PARP-1 overexpression. Induction of most DNA repair genes very early in the carcinogenic process was also observed by others [46]. This may be a part of a carcinogenic program where repair pathways in tumor cells may be upregulated to ensure their survival [21].

Concerning OGG1 and PARP-1 expression, the differences between the diseased tissue and normal one were more pronounced on the level of proteins than mRNA. Interestingly, the number of cells positively stained for PARP-1 and OGG1 was lower in adenoma tissues than in cancerous ones, although the intensity of staining was much stronger in polyps than in cancerous tissues (Fig. 3). One reason for the higher expression of PARP-1 and OGG1 proteins than mRNA may be the direct interaction between the proteins, which is enhanced as a result of oxidative stress [8]. It is possible that formation of such a complex may stabilize both proteins. Indeed, it has been found that binding of PARP-1 to the KLF8 protein enhanced KLF8 stability [47].

An intriguing association was found between OGG1 polymorphism and PARP-1 protein level. Several studies suggest that individuals carrying OGG1 326 Cys/Cys genotype have a higher risk of lung, prostate and nasopharyngeal cancer [48]–[51]. Our previous studies also indicated higher frequency of OGG1 326 Cys/Cys genotype among lung and colon cancer patients, and 8-oxoGua excision activity was lower in patients with only Cys variant in comparison to those with Ser/Cys or only Ser variants [11], [52]. This suggests that OGG1 326 Ser/Cys polymorphism may be a risk factor for developing some cancer types. Here, individuals bearing the *OGG1 Cys326Cys* genotype had a significantly higher PARP-1 level than Ser326Ser homozygotes or Ser326Cys heterozygotes (Fig. 5). At the moment it is difficult to explain such an association. Both proteins interact directly [8], upon interaction OGG1 stimulates the poly(ADP-ribosyl)ation activity of PARP-1. Polymorphic OGG1 form also binds to PARP-1 since interaction occurs *via* its unchanged N-terminal domain, but it is unable to activate PARP-1 [8]. It cannot be excluded that such abortive binding might exert an effect on PARP-1 stability.

Summing up, we have demonstrated high statistically significant correlation between PARP-1 and OGG1 and a much higher expression of both DNA repair proteins in diseased tissues in comparison with normal ones. It has been demonstrated that cells with OGG1 deficiency are more sensitive to PARP-1 inhibitors [8]. Therefore, our result that patients with the *OGG1 Cys326Cys* genotype had a significantly higher PARP-1 protein level than those with the *Ser326Cys* and *Ser326Ser* genotype may suggest that the diseased cells with polymorphic OGG1 recruit more PARP protein, which is necessary for removal of 8-oxodGuo. However, this needs further studies Thus, our findings may have some clinical implications since patients with decreased activity of OGG1/polymorphism of the OGG1 gene and higher 8-oxodGuo level may be more susceptible to PARP-1 inhibitors treatment.

## Supporting Information

S1 Fig **Lack of correlation between PARP-1 and OGG1 proteins in normal tissues.**
(DOCX)

S2 Fig **Western analysis of OGG1 protein (36 and 38 kDa) in colon tissues of CRC patients in relation to Lamin A/C (62 and 69 kDa).**
(DOCX)

S1 Table **Comparison of mRNA expression of PARP-1 and OGG1 in leukocytes.**
(DOC)

S2 Table **mRNA level of c-MYC in normal colon, adenomas and carcinomas.**
(DOCX)

S3 Table **Comparison of 8-oxodGuo level in leukocytes and tissues of AD and CRC patients in relation to healthy controls.**
(DOCX)
