# Supplementary material for: PARP-1 Expression is Increased in Colon Adenoma and Carcinoma and Correlates with OGG1
Source: PLoS One. 2014 Dec 19;9(12):e115558. doi: 10.1371/journal.pone.0115558 (PMC4272268; doi:10.1371/journal.pone.0115558)
Supplement: S1 Fig — Lack of correlation between PARP-1 and OGG1 proteins in normal tissues. (DOCX) [file pone.0115558.s001.docx]

**Figure S1**

**Lack of correlation between PARP-1 and OGG1 proteins in normal tissues**
