# Supplementary material for: PARP-1 Expression is Increased in Colon Adenoma and Carcinoma and Correlates with OGG1
Source: PLoS One. 2014 Dec 19;9(12):e115558. doi: 10.1371/journal.pone.0115558 (PMC4272268; doi:10.1371/journal.pone.0115558)

**Figure S2**

**Western analysis of OGG1 protein (36 and 38 kDa) in colon tissues of CRC patients in relation to Lamin A/C (62 and 69 kDa).**


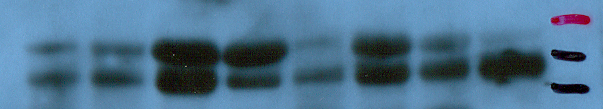


NG 058

NG056

NG038

NG022

NG019

NG011

NZ058

NZ013

Cys/Cys genotype

Ser/Ser genotype


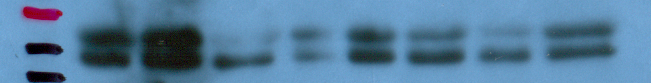


NG001

NZ030

NZ041

NZ047

NZ061

NZ062

NG062

NZ063


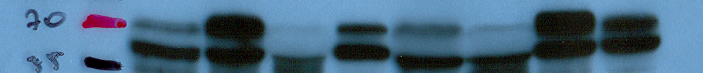


OGG1

Lamin A/C


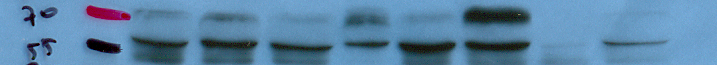

Supplement: S2 Fig — Western analysis of OGG1 protein (36 and 38 kDa) in colon tissues of CRC patients in relation to Lamin A/C (62 and 69 kDa). (DOCX) [file pone.0115558.s002.docx]
