# Supplementary material for: PARP-1 Expression is Increased in Colon Adenoma and Carcinoma and Correlates with OGG1
Source: PLoS One. 2014 Dec 19;9(12):e115558. doi: 10.1371/journal.pone.0115558 (PMC4272268; doi:10.1371/journal.pone.0115558)
Supplement: S1 Table — Comparison of mRNA expression of PARP-1 and OGG1 in leukocytes. (DOC) [file pone.0115558.s003.doc]

**Table S1**

**Comparison of mRNA expression of PARP-1 and OGG1­ in leukocytes**

| **Level of mRNA expression of PARP-1** | | | |
| --- | --- | --- | --- |
|  | **Healthy volunteers** | **Adenoma patients (AD)** | **Carcinoma patients (CRC)** |
| Mean  ±SD | 2.76  ±0.90 | 2.56  ±0.94 | 2.43  ±0.88 |
| Median  (range) | 2.72  (1.98-3.22) | 2.39  (2.02-2.91) | 2.37  (1.84-2.87) |
| **Level of mRNA expression of OGG1** | | | |
|  | **Healthy volunteers** | **Adenoma patients (AD)** | **Carcinoma patients (CRC)** |
| Mean  ±SD | 3.58  ±2.25 | 3.96  ±2.29 | 3.55  ±1.79 |
| Median  (range) | 3.31  (1.98-4.55) | 3.32  (2.43-4.52) | 3.02  (2.41-4.15) |
