# Supplementary material for: PARP-1 Expression is Increased in Colon Adenoma and Carcinoma and Correlates with OGG1
Source: PLoS One. 2014 Dec 19;9(12):e115558. doi: 10.1371/journal.pone.0115558 (PMC4272268; doi:10.1371/journal.pone.0115558)
Supplement: S2 Table — mRNA level of c-MYC in normal colon, adenomas and carcinomas. (DOCX) [file pone.0115558.s004.docx]

**Table S2.**

**mRNA level of c-MYC in normal colon, adenomas and carcinomas.**

|  | **Normal colon (N)** | **Adenoma**  **(A)** | **Carcinoma**  **(C)** | ***P*** |
| --- | --- | --- | --- | --- |
| **c-MYC mRNA** | **1.8147** | **5.1110** | **7.2112** | **0.00176 P *vs* N**  **0.00141 C *vs* N**  **0.10 P *vs* N** |
