# Supplementary material for: PARP-1 Expression is Increased in Colon Adenoma and Carcinoma and Correlates with OGG1
Source: PLoS One. 2014 Dec 19;9(12):e115558. doi: 10.1371/journal.pone.0115558 (PMC4272268; doi:10.1371/journal.pone.0115558)
Supplement: S3 Table — Comparison of 8-oxodGuo level in leukocytes and tissues of AD and CRC patients in relation to healthy controls. (DOCX) [file pone.0115558.s005.docx]

**Table S3**

**Comparison of 8-oxodGuo level in leukocytes and tissues of AD and CRC patients in relation to healthy controls.**

|  | **Level of 8-oxodGuo level/10^6^dG in DNA** | | | | | |
| --- | --- | --- | --- | --- | --- | --- |
|  | **Healthy group** | **AD patients** | | **CRC patients** | | |
|  | **Leukocytes**  **(N=121)** | **Leukocytes**  **(n=105)** | **Polyp tissue**  **(n=25)** | **Leukocytes**  **(n=136)*, **** | **Marginal tissue**  **(n=133) **** | **Tumor tissue**  **(n=133)*** |
| **Main** | 5.33 | 7.05 | 5.77 | 8.90 | 6.05 | 5.12 |
| **SD** | 2.30 | 2.87 | 2.42 | 4.95 | 3.80 | 3.12 |

***,** - p <0.05**
